# Case Reports

**Published:** 1900-01

**Authors:** G. E. Nesom

**Affiliations:** Clemson College, S. C.


### CASE REPORTS.

By G. E. Nesom, D.V.M.,
CLEMSON COLLEGE, S. C.

Case I.—An iron-gray mule, eight years old, weighed about 800 pounds. Brought to the College Veterinary Hospital by the owner, who had traded for the mule two years before. At that time he noticed a small open ulcer about the centre of the right anterior tibial region. This soon healed, but gradually enlarged for several months, having assumed the form of a base-ball under the skin. On examination the tumor proved quite hard, well defined, closely attached to the skin, and only slightly attached to the subjacent tissues. The mule was cast and secured with Conkey harness, hair clipped, and skin disinfected. An elliptical incision was made, and the remaining skin dissected back with some difficulty. This done the tumor was easily removed. The wound was washed with creolin solution, packed with boric acid, silk stitched, and the mule returned home, a distance of seven miles. The wound soon healed entirely, with no scar. On examination of the mass removed it showed to be the size of a small base-ball, with the flat side nearest the limb. It was composed almost entirely of bony matter, irregularly formed, and having a cavity much like a small brain cavity, filled with serum and globules of fatty matter. Specimen preserved.

Case II.—A small " clay-bank " mule, seven years old, weighed about 650 pounds, used for light farm work just at the foot of Blue Ridge Mountains. Brought to the college free clinic to have an enlargement on the side of the jaw " cut off."

The mass appeared just above the ring in the snaffle-bit on the right side; was some four inches long, tapering to the ends, and appeared one and a half inches in diameter. By placing the finger on Sterno's duct just above the margin of the jaw, and then moving the tumor, traction on the duct was easily detected. The mule was placed on the operating-table, part prepared, and a linear incision made straight down into the mass. After passing through the skin and a rather heavy layer of fibrous tissue the knife came in contact with a hard stone. By enlarging the opening to about one and a half inches a well formed calculus was removed. It was kidney-shaped, though more conical at the ends and measured three inches in length by one and three-eighth inches in diameter at the largest part. The dilated duct was stitched through with catgut so as to bring the mucous surfaces together, then the edges pared and stitched with silk. The skin, which had been dissected back, was closed with silk after being packed with boric acid. Do not know result of wounds.

Dr. Iago, of Athens, Ga., reports a traumatic injury of the lower end of the tibia, of more than a year's standing, caused by a kick; for three months previous had continually grown more lame. Surgical means for removal of the bony enlargement were adopted, and, though followed by a partially opened joint, in three months the parts had healed, the animal was placed at work, and there proved to be no stiffness of the hock articulations.

